# Autophagic response in the Rabbit Hemorrhagic Disease, an animal model of virally-induced fulminant hepatic failure

**DOI:** 10.1186/1297-9716-45-15

**Published:** 2014-02-04

**Authors:** Daniela Vallejo, Irene Crespo, Beatriz San-Miguel, Marcelino Álvarez, Jesús Prieto, María Jesús Tuñón, Javier González-Gallego

**Affiliations:** 1 Institute of Biomedicine (IBIOMED), University of León, 24071 León, Spain; 2 Centro de Investigación Biomédica en Red de Enfermedades Hepáticas y Digestivas (CIBERehd), Spain; 3 Department of Animal Health, University of León, 24071 León, Spain; 4 Division of Hepatology and Gene Therapy, Center for Applied Medical Research (CIMA), University of Navarra, Pamplona, Spain

## Abstract

The Rabbit Hemorrhagic Disease Virus (RHDV) induces a severe disease that fulfils many requirements of an animal model of fulminant hepatic failure. However, a better knowledge of molecular mechanisms contributing to liver damage is required, and it is unknown whether the RHDV induces liver autophagy and how it relates to apoptosis. In this study, we attempted to explore which signalling pathways were involved in the autophagic response induced by the RHDV and to characterize their role in the context of RHDV pathogenesis. Rabbits were infected with 2 × 10^4^ hemmaglutination units of a RHDV isolate. The autophagic response was measured as presence of autophagic vesicles, LC3 staining, conversion of LC3-I to autophagosome-associated LC3-II and changes in expression of beclin-1, UVRAG, Atg5, Atg12, Atg16L1 and p62/SQSTM1. RHDV-triggered autophagy reached a maximum at 24 hours post-infection (hpi) and declined at 30 and 36 hpi. Phosphorylation of mTOR also augmented in early periods of infection and there was an increase in the expression of the endoplasmic reticulum chaperones BiP/GRP78, CHOP and GRP94. Apoptosis, measured as caspase-3 activity and expression of PARP-1, increased significantly at 30 and 36 hpi in parallel to the maximal expression of the RHDV capsid protein VP60. These data indicate that RHDV infection initiates a rapid autophagic response, perhaps in an attempt to protect liver, which associates to ER stress development and is independent from downregulation of the major autophagy suppressor mTOR. As the infection continues and the autophagic response declines, cells begin to exhibit apoptosis.

## Introduction

The Rabbit Hemorrhagic Disease Virus (RHDV) is a positive-strand RNA virus, member of the *Caliciviridae* family, that causes in wild and domestic rabbits an acute highly fatal disease first reported three decades ago [1]. Hepatic damage plays a central pathogenic role and is histologically similar to fatal viral hepatitis causing fulminant hepatic failure (FHF) in humans [2]. We have shown by data on animal survival, clinical features, histopathological findings, changes in blood chemistry and intracranial pressure monitoring that the RHD fulfils many of the requirements of an animal model of FHF [3]. Moreover, loss of the oxidant/antioxidant balance [4], presence of apoptosis and endoplasmic reticulum (ER) stress [5-7], and lack of regeneration [8,9] are constant features in rabbits experimentally infected with the RHDV. This model could therefore be useful to improve our insight into the pathophysiology of viral FHF and to facilitate the development and evaluation of new therapeutic modalities.

Macroautophagy (thereafter referred to as autophagy) pathway is a bulk degradation system which controls the clearance and recycling of intracellular constituents for the maintenance of cellular survival [10] and can participate in the host response to infection [11]. Autophagy starts with the formation of a doubled-membrane-bound vacuole, known as the autophagosome, that engulfs fractions of the cytoplasm in an either unselective or selective manner via the activity of the autophagy adaptors, such as sequestrosome 1 (p62/SQSTM1), that forms a bridge between the target and the growing autophagosome membrane [12]. After being formed, most autophagosomes receive input from the endocytic vesicles to form an amphisome, in which the autophagic cargo is degraded and delivered into the lysosomal lumen [13]. The first step in the initiation of autophagy is the activation of a molecular complex containing the serine/threonine kinase ULK1. The activation of this complex is down-regulated by mammalian target of rapamycin (mTOR), which integrates multiple signalling pathways sensitive to the availability of amino acids, ATP, growth factors, or level of reactive oxygen species [14]. The nucleation of the autophagosomal membrane is controlled by another molecular complex containing Bcl-2-interacting protein (beclin)-1, which allows the production of phosphatidylinositol 3-phosphate (PI3P) to occur [15]. Several interacting proteins which participate in this complex include positive factors such as UV radiation resistance-associated gene (UVRAG) [16]. In the elongation step, two distinct ubiquitin-like conjugation systems are involved. The ubiquitin-like autophagy-related (Atg)12 is conjugated to Atg5 and then forms a complex with Atg16L1, which is required in the elongation of the autophagosome membrane and determines its curvature. The main player in the second conjugation system is microtubule-associated protein 1 light chain (LC)3, which is cleaved to generate the LC3-I form. LC3-I conjugates with phosphatidylethanolamine to LC3-II, which localizes to the autophagosomal membrane and is suited to serving as an autophagy-specific marker [10].

Autophagy primarily fulfills a pro-survival role during adaptation to unfavourable growth conditions or following cellular stress. In addition, autophagy contributes to innate immunity by degrading intracellular pathogens, and its inhibition results in an increased replication of virulence of different viruses such as herpex simplex virus 1 (HSV1) or vesicular stomatitis virus (VSV) [17]. However, many viruses, including hepatitis C virus (HCV), Dengue virus, or human immunodeficiency virus (HIV)-1, have evolved mechanisms to evade autophagy and in some cases manage to be even more subversive, using the autophagic response for enhanced replication and viral release [18,19]. ER stress, which is one of the typical stress responses initiated in cells after viral infection, is important in maintaining the physiology of healthy cells and functions to down-regulate protein synthesis through the unfolded protein response (UPR) [20]. It has been reported that autophagy is activated upon ER stress as a defensive mechanism for survival [21], and it is known that some viruses stimulate signalling pathways from UPR to autophagy [22]. Interference of the autophagic response with cell death mechanisms plays an important role in determining the fate of infected cells, and recent data suggest the existence of a cross talk between autophagic and apoptotic pathways [23]. For example some studies have demonstrated that the autophagy process can contribute to the death of virus-infected cells through apoptotic mechanisms [24]. However, the autophagy-dependent modulation of cell death is a complex phenomenon and it has also been reported that autophagy can prolong survival of virus-infected cells by counteracting the apoptotic response [25,26].

We have previously reported that RHDV leads to the activation of the different branches of the UPR [7] and induces apoptotic death in the last stages of the disease [5,6]. However it is unknown whether the RHDV induces autophagy in the liver of infected rabbits and how it relates to ER stress and apoptosis. In this study, we attempted to explore which signalling pathways were involved in the autophagic response induced by the RHDV and to characterize the role of autophagy in the context of RHDV pathogenesis.

## Materials and methods

### Virus and experimental model

Nine-week-old male New Zealand white rabbits were kept in the animal facility of the University of León with 12-h light cycle at 21–22 °C and 50% relative humidity. They were given a standard dry rabbit food and water *ad libitum*. Rabbits were injected intramuscularly with 2 × 10^4^ hemagglutination units of the RHDV isolate AST/89 [3,4] at 21 h pm. We have previously reported that during experimental RHDV-infection biochemical data and expression of genes involved in injury, apoptosis, ER stress and regeneration change remarkably at 36–48 hpi, with a 10-15% survival rate by 48 hpi [6-8]. So, we decided to study the effects of infection on the mechanisms of autophagy in rabbits that were infected with the RHDV and sacrificed at 12, 18, 24, 30 and 36 hpi (*n* = 6 each). The study was carried out in strict accordance with the recommendations in the Guide for the Care and Use of Laboratory Animals of the National Institutes of Health, and was specifically approved by the Ethics Committee of the University of León.

### Transmission electron microscopy

For transmission electron microscopy (TEM) analysis, liver tissues were dissected into 1-mm^3^ pieces for good penetration of the fixative, and then immersed in a modified Karnovsky fixative (2% glutaraldehyde + 4% buffered formalin (0.1 mol/L phosphate buffer)) overnight. The samples were post-fixed in 2% osmium tetroxide for 2 h at 4 °C and dehydrated with ascending grades of alcohol. The tissue block was then infiltrated and embedded in epon resin at 60 °C for 72 h. Ultrathin sections (70 nm) were cut with an automatic ultra-microtome (Reichert Ultracut E, Vienna, Austria) using a diamond knife. The sections were collected on copper grids (200 meshes) and stained with uranyl acetate and lead citrate solutions. TEM images were observed under a transmission electron microscope (JEOL Ltd, Tokyo, Japan) operating at an accelerating voltage of 80 kV.

### Real-time RT-PCR

Total RNA was extracted from frozen rabbit liver using a Trizol reagent (Life Technologies, Madrid, Spain) and quantified using Nano Drop1000 spectrophotometer (Thermo Scientific, Wilmington, DE, USA). Residual genomic DNA was removed by incubating RNA with RQ1 RNase-free DNase (Promega, Madison, WI, USA). RNA integrity was confirmed by formaldehyde gel electrophoresis. Total RNA (1 μg) was reverse transcribed as described [7] and mRNA was determined by real-time PCR analysis using SYBR Green I Master (Roche Diagnostics GmbH, Mannheim, Germany) and the appropriate primers (Table 1). Relative changes in gene expression levels were determined using the 2-DDCt method [27]. The cycle number at which the transcripts were detectable (Ct) was normalized to the cycle number of β-Actin gene detection, referred to as ΔCt.

**Table 1:** Primers used in this study

**Gene**	**Sense primer (5′-3′)**	**Antisense primer (5′-3′)**	**Accession number**	**Fwd start**	**Rev start**
Beclin-1	CATGCAATGGTGGCTTTCC	TCTCGCCCTTTTCAACCTCTT	XM_002719409.1	936	994
UVRAG	GCGGCGTCTTCGACATCT	GATGGCCGTTTCTATTAACAATGTT	XM_002708684.1	117	178
Atg5	CGTCCTGTGGCTGCAGATG	AAGGACACACTTCTTTGAGGAGATC	XM_002714882	417	479
Atg12	TGCTGAAGGCTGTGGGAGAT	TGTTCGCTCTACAGCCCATTT	XM_002712042.1	176	237
Atg16L1	CCACCAAACCGGCATGAG	CTTGCAGCTGGCTGTCATTC	XM_002721435.1	190	250
p62/SQSTM1	AACAGAGGTGACCACCCTTCA	AGCACAGACTGGCTGGAAGTC	XM_002710315.1	738	798
RHDV	TAGCCCAACAGAAGCACAAG	AAACAAGTCGTCAACCTCCC			
BiP	ATTGACAATGGTGTCTTCGAAGTC	CCCCGCCCAGGTGAGT	XM_002720437.1	709	766
CHOP	ATACATCACCACACCTGAAAGCA	GCACTCGGCTGCCATCTC	XM_002720915.1	103	160
GRP94	TGCTTAATTGGATGAAAGACAAA	GCTGAGACACCACAGCCTTTT	XM_002711230.1	1772	1834
β−Actin	TGGCATCCTGACGCTCAA	TCGTCCCAGTTGGTCACGAT	NM_001101683	262	317

### Western blot analysis

For Western blot analysis liver tissue (25 mg) was homogenised in 1 mL from RIPA buffer containing protease and phosphatase inhibitor cocktails (Roche Diagnostics GmbH). Further disrupt and homogenize tissue with a manual homogenizer, maintaining temperature at 4 °C throughout all procedures. Then the homogenate was incubated on ice for 30 min and finally the samples were centrifuged at 13 000 *g* for 30 min at 4 °C [28]. The supernatant fraction was recollected and stored at −80 **°**C in aliquots until use. Protein concentration was measured by Bradford assay. Equal amounts of protein extracts (30 μg) were separated by 7-12% sodium dodecyl sulphate (SDS)-polyacrylamide gel electrophoresis and transferred electrically to polyvinyllidene difluoride membranes (Millipore, Bedford, MA, USA). The membranes were then blocked with 5% non-fat dry milk in Tris-buffered saline containing 0.05% Tween 20 (TBST) for 30 min at 3 **°**C and probed overnight at 4 **°**C with polyclonal anti-p62/SQSTM1, PARP-1, Bcl-2, Bcl-xL (Santa Cruz Biotechnology, Santa Cruz, CA, USA), LC3I/II, and phospho-mTOR (Abcam, Cambridge, UK) antibodies at 1:200–1:1000 dilution with PBST containing 2.5% non-fat dry milk. Equal loading of protein was demonstrated by probing the membranes with a rabbit anti-GAPDH polyclonal antibody (1:2000 Sigma, St Louis, MO, USA). After washing with TBST, the membranes were incubated for 1 h at room temperature with secondary HRP conjugated antibody (Dako, Glostrup, Denmark, 1:5000), and visualized using ECL detection kit (Amersham Pharmacia, Uppsala, Sweden) [7]. The density of the specific bands was quantified with an imaging densitometer (Scion Image J Software 1.46a, Bethesda, MD, USA).

### Immunohistochemistry

Tissue samples were recovered, fixed in 10% buffered formalin and embedded in paraffin. Sections (4 μm) were dewaxed and hydrated through graded ethanol, cooked in 25 mM citrate buffer, pH 6.0, in a pressure cooker for 10 min, transferred into boiling deionized water and let to cool for 20 min. Tissue sections were then treated with 3% hydrogen peroxide to inactivate endogenous peroxidase activity. The slides were incubated with rabbit anti-VP60 and anti-LC3 antibodies (Ingenasa, Madrid, Spain and Abcam, respectively) overnight at 4 °C. Subsequently, the sections were incubated for 30 min using the EnVision + system and developed with a solution of 3-3-diaminobenzidine (DAB) (Vector Lab, Burlingame, CA, USA). The slides were stained with hematoxylin for 10 s and mounted. The specificity of the technique was evaluated by negative controls (omitting the incubation with the primary antibody and incubating it with non-immune sera). Pathological findings were assessed by one of the authors blinded to the group allocations.

### Caspase activity

Lysates were prepared by homogenizing liver tissue in 0.25 mM sucrose, 1 mM EDTA, 10 mM Tris and a protease inhibitor cocktail (Roche Diagnostics GmbH). The lysates were then centrifuged at 14 000 *g* for 10 min at 4 °C, and supernatants (50 μg protein) were incubated for 1 h at 37 °C in HEPES buffer containing 100 μM concentrations of the specific fluorogenic substrate (7-amino-4-methylcoumarin N-acetyl-L-aspartyl-L-glutamyl-L-valyl-l-aspartic acid amide, Ac-DEVD-AMC). Cleavage of the caspase substrate was monitored using a spectrofluorimeter (Hitachi F-2000 fluorimeter, Hitachi LTD, Tokyo, Japan) at excitation/emission wavelengths of 380/460 nm. Activity was expressed as fluorescence units per milligram of protein per min of incubation.

### Statistical analysis

Results are expressed as mean values ± standard error of the mean (SEM). Data were compared by analysis of variance (ANOVA); when the analysis indicated the presence of a significant difference, the means were compared with the Newman-Keul’s test. Significance was accepted when *p* was less than 0.05. Values were analyzed using the statistical package SPSS 19.0 (IBM Corporation, Armonk, NY, USA).

## Results

### Expression of the capsid protein VP60

**Figure 1:**
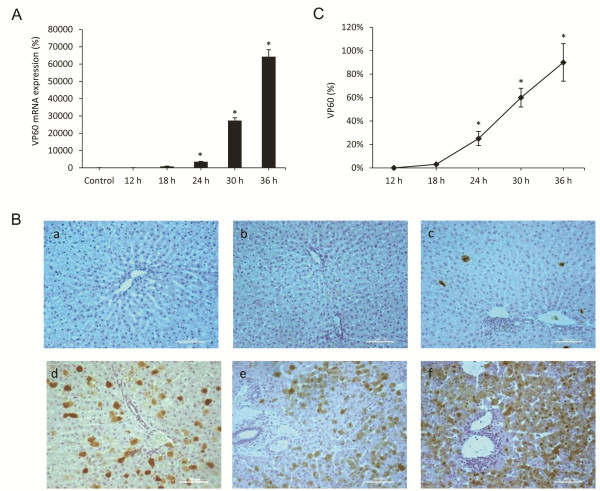
**Liver expression of the RHDV capsid protein VP60. A**: Levels of mRNA analyzed by real-time PCR assay and normalized against β-Actin. Data are presented as percentage change from the control group. Values are expressed as means SEM (*n* = 6). **B-C**: Immunohistochemical labeling in hepatocytes (a) Control, (b) RHDV 12 hpi; (c) RHDV 18 hpi; (d) RHDV 24 hpi; (e) RHDV 30 hpi; (F) RHDV 36 hpi. Paraffin-embedded sections were immunostained with a VP60 antibody. Original magnification: 200×. The graph shows evolution of the percentage of positively labeled hepatocytes over time. Values are expressed as means S.E.M (*n* = 6). *P < 0.05, compared with Control. Image analysis was performed using the ImageJ software v3.91 [30].

The RHDV is a single positive stranded RNA virus with a 40 nm icosaedric capsid composed by 90 dimers of the capsid protein VP60, and a minor structural protein VP2 which regulates capsid protein levels [29]. The expression level of the VP2 protein is very low and has been estimated to be one-fifth of that of VP60; therefore, to determine the presence of the virus in infected hepatocytes we employed VP60 mRNA as a viral marker, and its expression was analysed in liver extracts by quantitative real-time PCR (Figure 1A). As might be expected [29], the relative VP60 mRNA expression increased exponentially after 18 hpi in RHDV-infected rabbits. Viral VP60 antigen was also examined by immunohistochemical techniques too (Figure 1B-C). Labelling was not found in infected rabbits from the group of animals killed at 12 hpi. Viral VP60 antigen was first detected in hepatocytes from animals killed at 18 hpi. The extent of labelling increased markedly at 24 hpi, with the labelled hepatocytes mainly found in the periportal area. At 30 and 36 hpi, the liver revealed extensive viral VP60 antigen immunolabelling.

### Autophagy vesicles were detected in RHDV-infected hepatocytes by transmision electron microscopy

**Figure 2:**
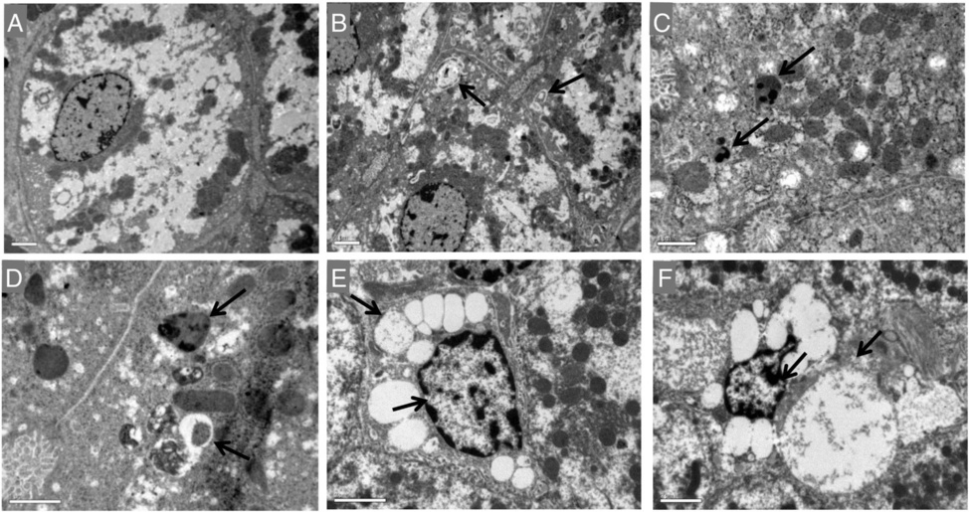
**Liver transmission electron microscopy of RHDV-infected rabbits. (A)** Control, **(B)** RHDV 12 hpi; **(C)** RHDV 18 hpi; **(D)** RHDV 24 hpi; **(E)** RHDV 30 hpi; **(F)** RHDV 36 hpi. Electron micrographs of the liver from control rabbits **(A)** and infected rabbits **(B-F)**. Normal appearance of hepatocytes was observed in rabbits from the control group **(A)**. Early disease periods **(B-D)** showed increased levels of lysosomes and mitochondria as well as an augmentation of their density. Images revealed a great number of autophagic vacuoles (black arrows) in different stages. In more advanced disease periods **(E-F)** the chromatin was condensed and aggregated at the periphery of the nuclear membrane and hepatocytes showed vacuolization of the cytoplasm (black arrows). Original magnification: 5000 – 15 000×.

One standard method to monitor autophagy is TEM, which together with immunohistochemical localization of LC3 enables the detection of autophagosomes. The TEM examination in this study reveals that autophagic structures were present in liver cells from 12 hpi (Figure 2). Phagophore structure, double-membrane autophagosomes with engulfed damaged organelles, and autolysosomes with a large vacuole containing large amount of cellular debris were present at 18 and 24 hpi. Severe cytoplasmic biliary necrosis was observed in the periportal rather than centrilobular hepatocytes, characterized by the accumulation of electron-dense biliary materials and markedly increased number of lysosomes. The bile canalicular microvilli were very swollen and stunted. Condensation of nuclear chromatin and cytoplasmic vacuolization, which is a typical sign of apoptosis, were observed at late infection periods, 30 and 36 hpi (Figure 2).

### RHDV infection induced autophagy molecular signalling

Monitoring static levels of autophagosomes is not sufficient to elucidate effects of RHDV on autophagy, because accumulation of autophagosomes may result either from an increased in their formation or a decrease in their fusion with lysosomes [31]. Thus, to examine autophagy in RHDV-infected rabbits and to avoid misinterpretation, in this research we combined the TEM study with immunohistochemical analysis, Western blot and RT-PCR of different autophagy markers.

**Figure 3:**
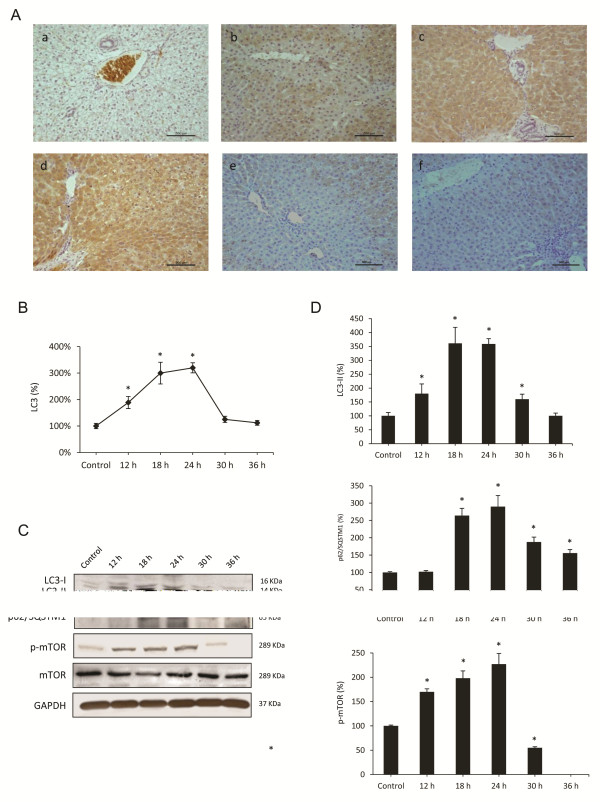
**Effects of RHDV infection on markers of autophagy. A-B**: Immunohistochemical labeling of the autophagy marker LC3 in the liver of RHDV-infected rabbits. (a) Control; (b) RHDV 12 hpi; (c) RHDV 18 hpi; (d) RHDV 24 hpi; (e) RHDV 30 hpi; (F) RHDV 36 hpi. Paraffin-embedded sections were immunostained with a LC3 antibody. Original magnification: 200×. The graph shows evolution of the percentage of positively labeled hepatocytes over time. Values are expressed as means S.E.M (*n* = 6). *P < 0.05, compared with Control. Image analysis was performed using the ImageJ software v3.91 [30]. **C-D**: Western blot of markers of autophagy. Proteins from liver extracts were separated by sodium dodecyl sulfate polyacrylamide gel electrophoresis, followed by immunoblotting. Equal loading of proteins is illustrated by GAPDH bands. The graph shows densitometric quantification. Values are expressed as means S.E.M (*n* = 6). *P < 0.05, compared with Control.

LC3 is a major marker of the autophagosome formation and a protein widely used as a hallmarker of autophagy [32]. As shown in Figure 3A-B, immunoreactivity for LC3 was negative in liver sections from control rabbits. LC3 antigen was detected in hepatocytes as soon as 12 hpi. At 18 hpi LC3 immunolabelling increased significantly, reaching a peak at 24 hpi. At 30 and 36 hpi, hepatic sections revealed a decrease in the number of labelled hepatocytes. Stressors, such as some viruses, upregulate LC3 expression and promote the binding of cytosolic LC3-I to PE to form autophagosome-specific lipidated form LC3-II, which remains attached to the inner membrane, making it a good marker of autophagosomes. Thus, the conversion of LC3-I to LC3-II is a certain and specific marker of autophagy and necessary for the autophagosome formation [10]. When liver homogenates were analyzed by Western blot to detect the different forms of LC3, a significant increase in protein expression of LC3-II was observed at 18 and 24 hpi, with a later decrease at 30 and 36 hpi (Figure 3C-D). The results from TEM studies, LC3 hepatocyte labelling and LC3-II protein expression unequivocally demonstrate that the autophagy was induced at an early stage in rabbits infected with the RHDV.

**Figure 4:**
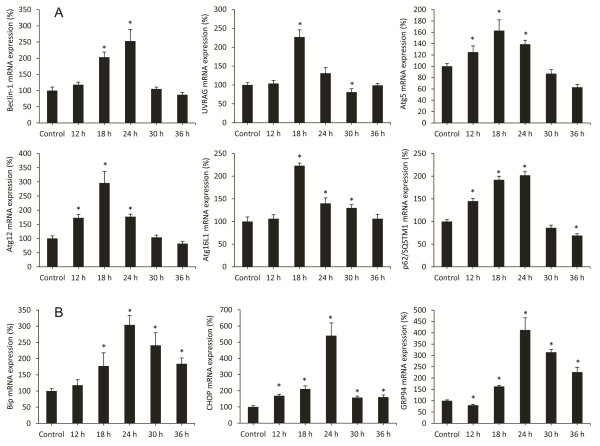
**Effect of Rabbit Hemorrhagic Disease virus (RHDV) infection on mRNA levels of genes related to autophagy (A) and ER stress (B).** Levels of mRNA were analyzed by real-time PCR assays. Data, normalized against β-Actin, are presented as percentage change from the control group. Values are expressed as means S.E.M (*n* = 6). *P < 0.05, compared with Control.

In addition to the LC3 system there is a second ubiquitine-like system essential for autophagosome formation which is formed by the Atg12-Atg5-Atg16L1 complex, which is situated in the outer layer of the isolation membrane [33]. To confirm that RHDV infection triggers autophagy activation we quantified mRNA expression of the complex components at different infection periods. Results obtained indicate that mRNA levels increase from 12 hpi for Atg12 and Atg5 and from 18 hpi for Atg16L1, reach a maximum at 18 hpi, and still remain significantly elevated at 24 hpi; values return to basal levels or even lower at 30 and 36 hpi (Figure 4). The beclin-1-PI3K complex is a critical element in the autophagy signalling pathway [15]. We observed that beclin-1 mRNA levels increased at 18 and 24 hpi with a decrease in later periods, in parallel to the changes detected in both ubiquitine-like systems (Figure 4). Beclin-1-PI3K -mediated autophagy is positively regulated by UVRAG, which interacts with beclin-1 in the early steps, leading to activation of autophagy through the autophagosome maturation [16]. UVRAG mRNA expression revealed a peak at 24 hpi coinciding with changes in beclin-1 mRNA expression, and then began to decrease. We further studied specific autophagy substrate p62/SQSTM1, an adaptor protein which plays an essential role in mediating selective autophagy, and serves as an autophagy receptor targeting ubiquitin proteins to autophagosomes for degradation [12]. p62/SQSTM1 mRNA and protein expression increased from 12 to 24 hpi, with decreases at 30 and 36 hpi (Figures 3C-D, 4).

### Effects of RHDV infection on pathways regulating autophagy induction

One of the major pathways regulating autophagy involves mTOR. It is known that activation of mTOR in nutrient-proficient cells acts as a negative regulator of autophagy, while repression of mTOR by nutrient deprivation or rapamycin treatment induces autophagy [14]. However, the cross talk between mTOR pathway and autophagy induction during viral infection is complex, and it has been reported that some viruses activate mTOR signalling [23,34]. We analyzed the hepatic expression of phospho-mTOR by Western blot at different RHDV-infection periods (Figure 3C-D). A progressive increased hepatic expression of phospho-mTOR was observed at 12, 18, and 24 hpi in RHDV-infected animals. However, at 30 hpi phospho-mTOR hepatic level decreased to values below the control group, and it was undetectable at 36 hpi.

Although the role of autophagy in normal ER function is not established, there are some studies that have shown that autophagy is associated with the ER and maybe an important part of normal ER function [21]. ER stress-induced autophagy plays an important role in maintaining cellular homeostasis through alleviating stress and can also be used as an alternative degradation mechanism to process misfolded proteins that have accumulated in the ER lumen [7]. During ER stress different transcription factors regulate the expression of ER chaperones that enhance the folding capacity of the ER, including CCAAT/enhancer-binding protein homologous protein (CHOP), immunoglobulin-heavy-chain-binding protein (BiP/GRP78) and glucose-regulated protein 94 (GRP94). BiP is an ER chaperone protein which is required for protein folding and has been recently shown to play a central role modulating the sensitivity and duration of the UPR [35]. Hepatic expression of BiP was measured by RT-PCR (Table 2). Results showed a progressive increase in the values at different time infection periods until 24 hpi. Activation of UPR in infected rabbits was confirmed by quantification of the mRNA level of CHOP, a major marker of the ER stress response, and GRP94, a molecular chaperone and resident protein of the ER that aids in the folding of secretory and membrane proteins [7]. Results showed a peak of mRNA expression for both chaperones at 24 hpi (Table 2).

**Table 2:** Effect of Rabbit Hemorrhagic Disease virus (RHDV) infection on mRNA levels of genes related to autophagy

	**Control**	**12 h**	**18 h**	**24 h**	**30 h**	**36 h**
Beclin-1	100 ± 11	118 ± 8	203 ± 16^*^	253 ± 36^*^	105 ± 6	87 ± 7
UVRAG	100 ± 6	104 ± 8	227 ± 19^*^	131 ± 15	81 ± 9^*^	99 ± 5
Atg5	100 ± 5	125 ± 11^*^	163 ± 19^*^	139 ± 7^*^	87 ± 7	63 ± 5
Atg12	100 ± 9	173 ± 12^*^	296 ± 41^*^	177 ± 9^*^	104 ± 8	82 ± 7
Atg16L1	100 ± 10	106 ± 9	223 ± 6^*^	140 ± 12^*^	130 ± 7^*^	106 ± 10
p62/SQSTM1	100 ± 4	145 ± 6^*^	192 ± 8^*^	202 ± 8^*^	86 ± 6	69 ± 4^*^
BiP	100 ± 7	118 ± 17	177 ± 41^*^	304 ± 29^*^	241 ± 39^*^	184 ± 18^*^
CHOP	100 ± 8	169 ± 10^*^	211 ± 19^*^	540 ± 79^*^	158 ± 9^*^	161 ± 13^*^
GRP94	100 ± 4	80 ± 4^*^	163 ± 5^*^	412 ± 54^*^	314 ± 13^*^	226 ± 21^*^

Levels of mRNA were analyzed by real-time PCR assays. Data, normalized against β-Actin, are presented as percentage change from the control group. Values are expressed as means±S.E.M (n = 6). *P<0.05, compared with Control.

### Apoptotic death in RHDV-infected liver cells

**Figure 5:**
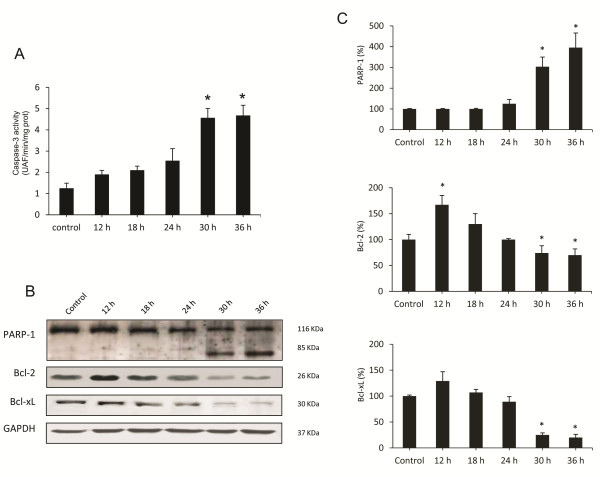
**Effects of RHDV infection on markers of apoptosis. A**: Liver activity of caspase-3. Values are expressed as means S.E.M (*n* = 6). *P < 0.05, compared with Control. Samples were incubated with the specific fluorigenic substrate Ac-DEVD-AMC. **B-C**: Western blot of markers of autophagy. Proteins from liver extracts were separated by sodium dodecyl sulfate polyacrylamide gel electrophoresis, followed by immunoblotting. Equal loading of proteins is illustrated by GAPDH bands. The graph shows densitometric quantification. Values are expressed as means S.E.M (*n* = 6). *P < 0.05, compared with Control.

Autophagy has a complex interaction with apoptosis. It can inhibit or cause cell death, and, on the other hand, apoptosis is known to inhibit the genesis of autophagy [36]. Moreover, it is know that autophagy plays a major role in determining the fate of virally infected cells by blocking or promoting apoptotic mechanisms [23]. We have previously reported that apoptotic liver damage develops in rabbits infected by the RHDV and the effect is attenuated by treatment with antioxidants [5,6]. In this study we analyzed changes with time in the activation of caspase-3, the common event initiated by multiple different stimuli that induces apoptosis [37]. Samples were incubated with a specific fluorigenic substrate whose cleavage indicated that infection resulted in a marked increase of caspase-3 activity only at 30 and 36 hpi (Figure 5A). Furthermore, Western blot analysis demonstrated that at later periods of infection there was a marked proteolysis of PARP-1 (Figure 5B-C), a nuclear enzyme whose cleavage into a 85-kDA fragment by caspase-3 confirms that cells are undergoing apoptosis [5]. Our data also show that at 30 and 36 hpi there is a significant inhibition of the expression of Bcl-2 and Bcl-xL (Figure 5B-C), two antiapoptotic proteins involved in the intrinsic pathway of apoptosis [6].

## Discussion

This research examined the occurrence of autophagy during experimental infection by the RHDV. Similarly to other studies conducted with viruses that promote autophagy, TEM analysis showed that number and content of autophagy vesicles increased in RHDV-infected livers. We further analyzed the impact of RHDV infection of several proteins that regulate distinct molecular events leading to autophagy vesicle formation, including its initiation (beclin-1), and maturation by the Atg12 and LC3 conjugation systems. Data obtained demonstrate an early increased expression of the Atg16L1 complex components, together with enhanced LC3 immunostaining and conversion of soluble cytosolic LC3-I to its lipidated, autophagosome-associated form LC3-II, which unequivocally demonstrates that the autophagy was induced at an early stage in rabbits infected with the RHDV. Real-time PCR confirmed that the key autophagy gene beclin-1 was also activated, a fact which suggests a crucial role for this protein in the induction of the autophagic response by the RHDV. Although beclin-1 up-regulation is a frequent finding following viral infection [13], there are data of beclin-1-independent autophagy induction by enterovirus 71 [38] and it has been reported a late and rather limited increase in the expression of this proautophagic protein by HSV-1 [26].

In our experiments, p62/SQSTM1 expression increased from 12 hpi and remained elevated until 24 hpi. p62/SQSTM1 is a multifunctional protein, involved in the delivery of ubiquitin-bound cargo to the autophagosome, that interacts with LC3 and is specifically degraded by the autophagic-lysosome pathway, being commonly measured to detect autophagic flux [12]. Viral infection with different herpes viruses has been reported to result in a decrease of p62/SQSTM1 in parallel to increase in the protein LC3-II [39,40]. However, upregulated expression of both p62/SQSTM1 and LC3 has been shown to exist in different types of tumours, whose growth is significantly inhibited by p62/SQSTM1 down-regulation [41]. Moreover, the expression of p62/SQSTM1 and LC3-II also increases in livers from patients with primary biliary cirrhosis and cultured biliary epithelial cells treated with hydrogen peroxide, with an accumulation of p62-positive aggregates [42]. In Huh 7.5 cells it has been reported that after the transfection of the HCV RNA there is a continuous increase of p62/SQSTM1 which indicates that HCV does not enhance autophagic protein degradation [43]. Results from the present research suggest a similar response to RHDV infection, with an upregulation of p62/SQSTM1 which may reflect a dysfunctional process in which the capacity of autophagy is not much enough to process the damaged proteins bound to p62/SQSTM1.

mTOR is an important signalling molecule which in nutrient-proficient cells acts as a negative regulator of autophagy [26]. When the expression of phospho-mTOR was monitored by Western blot assay we observed an increased expression between 12 and 24 hpi, showing that infection with the RHDV stimulates the mTOR signalling pathway in parallel to the development of the autophagic process. A similar unexpected result has been previously reported in HCV-infected human hepatocytes [34], in U251 glioma cells after infection with the Newcastle virus [44], and in bovine kidney cells infected with the bovine herpesvirus type-4 [40]. Our data demonstrate that mTOR is not a negative regulator during RHDV-induced autophagy, and could indicate that induction of autophagy occurs upstream of mTOR signalling or that both processes act concurrently. In HCV-infected hepatocytes it has been suggested that mTOR activation is necessary for cell growth through regulation of phospho-eukaryotic translation initiation factor 4E-binding protein (EBP)1 [34]. Further work would be necessary to identify if there is a similar requirement following infection with the RHDV.

Autophagy is also triggered in response to ER stress through the induction of the UPR [45]. In mammalian cells, knockdown of the upstream UPR regulator BiP inhibits autophagosome formation, but does not affect the conversion of LC3-I to LC3-II, suggesting that ER stress induction is an obligatory factor for autophagy and may function at the phagophore expansion rather than induction step [46]. Previous studies have shown that induction of autophagosomes by the HCV virus depends on the UPR [43], and the three branches of the UPR contribute to regulate HCV replication via modulation of autophagy [22]. It has also been reported that the tobacco mosaic virus RNA induces ER stress-related autophagy in HeLa cells [47], and it is known that autophagosome formation during varicella-zoster virus infection follows ER stress and the UPR [48]. In a previous work, our research group, using the RHDV model of FHF, reported that ER stress was induced in RHDV infected rabbits through a modulation of the three branches of the UPR [7]. Here, it is shown that mRNA levels of the molecular chaperones CHOP, BiP and GRP94 reached a peak at 24 hpi, in parallel to the increase of the expression of beclin-1 and the components of the two ubiquitin-like conjugation systems Atg12-Atg5-Atg16L1 and LC3. Our data suggest that autophagy could be provoked at least in part upon ER stress. This hypothesis is further supported by the RHDV-induced increase in the upregulation of beclin-1, whose expression is required for ER-stress induced autophagy [46].

The interplay between autophagy and programmed cell death is complex. Autophagy is a cytoprotective mechanism which enables cells to survive unfavourable growth conditions and can prevent cell death by apoptosis. However, some studies have demonstrated that autophagy may have an active contribution to cell death in virus infected cells. Thus, it has been reported that pharmacological inhibition of autophagy efficiently suppresses apoptosis induced by human adenovirus type 5 Delta-24-RGD mutant in mouse fibroblast or human U251 glioma cells [49]. Blocking of autophagy also attenuates cell death caused by the avian influenza A H5N1 virus both in vitro and in vivo [50], and it is known that knockdown of beclin-1 or Atg5 protects human rhabdomyosarcoma cells from enterovirus 71-induced apoptotic death [24]. We and others have previously reported that RHDV infection induces in rabbits a marked apoptotic response at 36–48 hpi with increased caspase-3 activity and immunoexpression and a marked proteolysis of PARP-1 [5,6,8,51]. Results from the present study indicate that apoptosis is present in the late stages of the disease, with no significant increase in caspase-3 activity and PARP-1 degradation or decreased expression of the antiapoptotic proteins Bcl-2 or Bcl-xL occurring in early periods. The fact that autophagy develops in hepatocytes at early stage and cells begin to exhibit apoptosis in parallel to the decline of the autophagy response, suggests that autophagy play a beneficial role in an attempt to protect cells from the impending noxious effects of the virus. It has been recently shown that cardiomyocites exposed to angiotensin II exhibit a similar behavior, with autophagy occurring at early stages whereas apoptosis occurs late [36]. A number of studies have also demonstrated the ability of virally-induced autophagy to prevent or delay death of infected cells. For example, apoptotic death of hepatoma cells expressing the oncogenic HBV X protein increases when autophagy is blocked [52], and the infection with Japanese encephalitis virus increases caspase activation and cell death in beclin-1 or Atg5-deficient cells [53]. It has also been shown that in HSV-1-infected U251 glioma cells the autophagic response markedly delayed caspase activation and other hallmarks of apoptotic cell death [26]. Data here obtained also point to a role of virally-induced autophagy to support survival of the infected cells and suggest that autophagy might contribute to limit the pathological consequences associated with cell death triggered by the RHDV infection. Confirmation of the connection between autophagy and RHDV pathogenecity should require the use of cell culture systems, that are unavailable at present [54]. An additional interesting finding concerns the increased expression of p62/SQSTM1 observed in liver cells. This reflects inhibition of autophagosome-lysosomal function and dysfunctional autophagy, which may contribute to altered signal transduction pathway and liver damage [55]. In fact, it is known that upregulation of p62/SQSTM1 positively controls apoptosis by polyubiquitination and aggregation of the key initiator caspase 8 [56,57], thus playing a potential role in the cross-regulation between autophagy and apoptosis.

In summary, experiments here reported were aimed to enhance our understanding of the interplay between the RHDV and the host liver cells. The most important finding is that RHDV infection in vivo initiates a rapid autophagic response, perhaps in an attempt to protect liver, which associates to ER stress development and is independent from down-regulation of the major autophagy suppressor mTOR. As the infection continues and the autophagic response declines, the process of apoptosis dominates. Although it is necessary to be cautious, considering that autophagy is also involved in modulation of viral replication and recognition/presentation of viral antigens [26], therapeutic potential of autophagy modulation in controlling RHDV-induced cell death is worthy to be explored, considering the importance of RHDV infection as a model of human FHF of viral origin. Findings from the present study could contribute to the search for new pharmacological strategies to protect livers from FHF injury.

## Competing interests

The authors declare that they have no competing interests.

## Authors’ contributions

DV, IC, MA and BS carried out the experiments. JP, JGG and MJT interpreted the results and contributed to the discussion. MJT and JGG were responsible for overall supervision. All authors read and approved the final manuscript.
